# Quality of Life in Children with Neurofibromatosis Type 1: Agreement between Parents and Patients, and the Role of Disease Severity and Visibility

**DOI:** 10.3390/children11081033

**Published:** 2024-08-22

**Authors:** Nicola Davide Cavallo, Paola Maietta, Silverio Perrotta, Pasquale Moretta, Marco Carotenuto, Maria Esposito, Gabriella Santangelo, Claudia Santoro

**Affiliations:** 1 Department of Psychology, University of Campania “Luigi Vanvitelli”, 81100 Caserta, Italy; 2 Child and Adolescent Neuropsychiatry Clinic, Department of Mental and Physical Health and Preventive Medicine, University of Campania “Luigi Vanvitelli”, 80138 Naples, Italy; 3 Referral Centre of Neurofibromatosis, Department of Woman and Child, University of Campania “Luigi Vanvitelli”, 80138 Naples, Italy; 4 Istituti Clinici Scientifici Maugeri IRCCS, Neuromotor Rehabilitation Unit of Telese Terme Institute, 82037 Benevento, Italy

**Keywords:** NF1, neurofibromatosis type 1, quality of life, QoL, parents’ report, disease visibility, disease severity

## Abstract

Background: Neurofibromatosis type 1 (NF1) is a genetic disorder that affects multiple systems in the body, often leading to physical disfigurements and a wide range of clinical symptoms. This study aims to investigate the relationship between NF1 severity and visibility and the quality of life (QoL) in children. Methods: The Pediatric Quality of Life Inventory (PedsQL) and a modified version of the Ablon scale were used to assess QoL and NF1 severity and visibility, respectively. Self-reported and parent-reported QoL scores were compared, and the associations between NF1 severity/visibility and QoL were explored. Results: Thirty-eight pediatric NF1 patients and their parents were enrolled. QoL scores did not differ significantly between patient self-reports and parent reports. However, correlational analyses revealed that higher NF1 severity was associated with lower physical QoL in patients, and greater NF1 visibility was linked to lower physical and social QoL. For parents, higher NF1 severity correlated with lower school functioning, whereas NF1 visibility did not show a significant correlation with QoL. Conclusion: The severity and visibility of NF1 have distinct impacts on various aspects of QoL in children, highlighting the need for tailored interventions that address both physical and psychological challenges. These findings underscore the importance of comprehensive care approaches in managing NF1 in pediatric populations.

## 1. Introduction

Neurofibromatosis type 1 (NF1) is an autosomal-dominant syndrome caused by mutations in the NF1 tumor-suppressor gene on chromosome 17 [1]. The incidence at birth is 1:2000–3000 individuals in the general population [2]. Approximately half of the cases are inherited, while the other half result from de novo mutations. NF1 is a multisystem disorder characterized by several dermatological manifestations, including café-au-lait macules (CALMs), cutaneous and plexiform neurofibromas (CNs and PNs) and Noonan-like features [2]. Patients often experience itching and pain, with the latter mainly associated with PNs. NF1 can also lead to skeletal deformities, short stature, learning difficulties and brain gliomas, which predominantly occur in young patients. The spectrum of potential physical, cognitive, psychological and social effects associated with NF1 is sufficiently broad to have a significant impact on an individual’s quality of life (QoL) [3,4,5,6]. While the relationship between NF1 and QoL in adulthood is well-documented, data on pediatric QoL remain scarce. Evidence from the literature suggests that all aspects of QoL (i.e., patients’ social and relational interactions, mental health, appearance, pain levels and daily activities) seem to be impaired [7,8,9]. The diminished QoL in NF1 appears to be due to the chronic nature, multisystem involvement and esthetic discomfort [10,11]. 

Regarding the visibility and dermatological manifestations of NF1, evidence indicates that a higher total number of cNFs or cNFs primarily located on the face are associated with worse QoL when assessed using skin disease-specific measures [9,12]. 

Some studies have shown that even in children, in whom cNFs are usually absent [2], there is a perceived visibility of NF1 that negatively affects their QoL [5,13]. Recently, we reported that, in a group of children and adolescents with NF1, greater visibility of NF1 symptoms is associated with lower self-esteem [14]. 

In addition to the visibility, the severity of NF1 symptoms is a fundamental determinant of patients’ QoL, psychosocial well-being and need for support in daily living [15,16,17]. Pain is a significant factor in the estimation of the severity of NF1 and can further degrade QoL [18,19,20]. Despite the significant impact of visibility and severity on QoL, research in this area remains limited.

When assessing pediatric populations, parents are an important source of information and are often asked about their children’s QoL [21]. The Pediatric Quality of Life Inventory (PedsQoL; ref. [22]) is a multidimensional questionnaire designed to assess the impact of illness on QoL. It includes a self-report version for patients and a proxy report for parents. The degree of agreement between patient and parent reports of QoL varies across studies, with discrepancies noted in all areas of the scale, including emotional, social and physical functioning [23,24]. 

It is plausible that the presence of neurofibromas, scoliosis and other visible manifestations associated with NF1 may affect the physical aspect of QoL [10]. Conversely, cognitive impairments, including intellectual disability, learning disabilities and executive deficits, which are quite common in patients with NF1, can affect school performance and social interactions, leading to lower social QoL [25,26]. 

In this study, we reanalyzed our previously published data on pediatric QoL in patients with NF1 using a modified Ablon scale [14], which includes more detailed features than the original scale [27]. Our aim was to examine the impact of visibility and severity on various domains of QoL and to explore the degree of agreement between children and their parents. We hypothesized that parents and patients would agree on QoL assessments and that NF1 visibility and severity would correlate with QoL. Specifically, we hypothesized that visibility would be associated with the socio-relational functioning of QoL.

## 2. Materials and Methods

### 2.1. Subjects and Procedure

The participants were children and adolescents diagnosed with NF1, recruited at the Pediatric Neurofibromatosis Referral Centre of the University of Campania “Luigi Vanvitelli”. Eligibility criteria included the following: (i) a confirmed diagnosis of NF1 based on the revised criteria established by Legius and colleagues [27]; (ii) no intellectual disability as defined by the DSM-5 [28]; and (iii) absence of other neurological or disabling conditions. Participants underwent a pediatric evaluation, followed by a psychological assessment. For this study, the PedsQoL [22], both patient self-report and parent report, and the modified Ablon scale [14] were administered to a previously studied cohort of pediatric patients with NF1 (for inclusion criteria, see [14]).

Two experienced clinicians independently assessed the severity and visibility of NF1 in each patient, and any discrepancies were resolved through discussion. Written informed consent was obtained from the custodial adults and the children, with assurances provided regarding the voluntary nature of participation and the confidentiality of the data collected. No participants withdrew from the study. 

The study, conducted in 2019, was performed in accordance with the ethical standards of the Declaration of Helsinki and its subsequent amendments; it was also approved by the Ethics Committee of the University of Campania “Luigi Vanvitelli”. 

### 2.2. Modified Ablon Scale

In the study by Cavallo et al. (2023) [14], we proposed a modified version of the Ablon scoring system [29]. Our clinical practice with patients led us to modify the original Ablon scale to better reflect the expanded understanding of the nature, impact and frequency of the numerous multisystemic features present in NF1 patients, particularly in children.

In the original Ablon scale, the visibility dimension assesses the extent of disfigurement caused by NF1-related neurofibromas and other associated features, such as café-au-lait spots and skin fold freckling. Visibility ratings are based on the appearance of the fully clothed person and the ease with which symptoms could be perceived in interpersonal interactions. In contrast, severity ratings are based on a combination of clinical and cosmetic implications that could potentially affect lifestyle, mobility and/or threaten life.

To account for the frequent absence of visible signs of NF1 during a casual inspection in childhood, we incorporated the rating “none” into the visibility dimension of our modified Ablon scoring system (see Table 1). This addition is crucial as several patients do not show neurofibromas or dysmorphic features even in adulthood.

We proposed that café-au-lait macules and freckles might be insignificant in both impersonal and interpersonal interactions. Furthermore, we included language deficits in the mild severity dimension, and in the moderate severity dimension, we added moderate scoliosis, other abnormal skeletal features, short stature and dysmorphisms. In this modified Ablon scale, severity ranges from mild to severe, while visibility ranges from absent to severe. The criteria for determining the severity and visibility of NF1 in both the modified version and original versions of the Ablon scale are shown in Table 1. 

### 2.3. Statistical Analysis

Due to significant violations of the normality assumption (Shapiro–Wilk *p*-value < 0.05), non-parametric statistical methods were employed. 

A two-tailed Mann–Whitney U-test was performed to compare the parent and self-report scores on QoL. Each subscale of the PedsQL (i.e., physical functioning, emotional functioning, social functioning, school functioning and QoL total score) was analyzed separately to investigate the impact of NF1 on different areas of daily life. In addition, to explore the potential association between QoL (self-report and parent report) and the severity and visibility of NF1, Spearman’s correlational analysis was conducted. The analyses were conducted utilizing IBM SPSS-20, with the critical alpha level set at 0.05.

## 3. Results

The study included 38 consecutive subjects with NF1 (median age = 14.07 years, range = 8–17 years, interquartile range [IQR] = 5.94 years; female *n* = 17) and their parents [14]. According to the modified Ablon scale, these patients were categorized based on the severity and visibility of their conditions. 

Specifically, in terms of severity, 5 patients (13.2%) had a severe disease, 19 (50%) had a moderate disease and 14 (36.8%) had a mild disease.

In terms of visibility, 3 patients (7.9%) had a severe phenotype, 22 (57.9%) had a moderate phenotype, 6 (15.8%) had a mild phenotype and 7 (18.4%) children had no visibility of their condition.

There was no significant difference between patient and parent reports on QoL (Table 2). However, a borderline statistically significant difference was observed in the social functioning domain (Table 2). 

In the patient group, the correlational analysis revealed that the severity score was significantly associated only with a lower physical QoL (r_s_ = 0.423, *p* = 0.009). However, the visibility score was associated with decreased physical (r_s_ = 0.416, *p* = 0.010) and social QoL (r_s_ = 0.338, *p* = 0.041). 

Conversely, within parent reports, severity was only related to lower school functioning (r_s_ = 0.385, *p* = 0.021), while visibility showed no significant association with the total score or any QoL subdomains (r_s_ = 0.215, *p* = 0.208). Finally, no significant associations were observed between the dimensions of quality of life (i.e., physical, emotional and social functioning) and the severity or visibility of NF1 in parents (r_s_ = 0.220, *p* = 0.191).

## 4. Discussion

The objective of this study was to compare the self-reported QoL of pediatric patients with the QoL reported by caregivers in relation to their children. To this end, we investigated both how pediatric patients assess their own QoL and how parents evaluate the QoL of their children. Our findings indicate agreement between parents and children in terms of overall QoL as well as in the specific domains of PedsQoL. Previous research has reported discordance between self-reported and proxy-reported QoL in children with NF1, suggesting that information from parents and patients may originate from different perspectives and should be considered together [21,30]. Nevertheless, some studies on chronic health pediatric conditions have shown a high degree of concordance between parent and child reports, particularly in more observable domains such as physical functioning, as observed in our cohort [21,31]. Discrepancies in the literature may be attributed to several factors impacting agreement levels, including the child’s age, the QoL domains examined, the severity of the children’s illness, the sociocultural background and the QoL of parents themselves [21,32,33]. If our results are confirmed by larger studies, parents could be considered valuable sources for describing the impact of the disease on QoL.

Additionally, we investigated the impact of NF1 severity and visibility on the various QoL domains. According to patient reports, greater NF1 severity was associated with poorer physical QoL, whereas greater NF1 visibility was linked to poorer physical and social QoL [30,34,35]. 

As widely demonstrated in the literature, the visibility of NF1 can impact QoL and mental health [4,11], as well as psychosocial aspects of QoL [15]. This relationship has also been observed in other diseases with visible manifestations [7,36]. Individuals with NF1 often face social stigma due to the visible manifestations of the disease, such as cNF and café-au-lait spots [7,37]. Such visibility can lead to stress, social anxiety, depression, reduced self-esteem and social isolation [14,19,38]. These individuals might experience anticipatory anxiety about social interactions, worrying about how others will react to their appearance. This persistent stress can contribute to mood disorders, creating a cycle where the psychological burden of the disease exacerbates the individual’s mental health [7]. Our results confirm that disease visibility interferes with social functioning and relationships in pediatric patients with NF1, similar to other visible medical conditions. This interference can lead to a diminished QoL and fewer opportunities for social support, which is crucial for mental well-being [39,40]. Patients with more prominent physical manifestations (i.e., visible symptoms) experience higher levels of social avoidance and stigmatization [41,42,43]. Children and adolescents with NF1 may appear more withdrawn [38,44], face socio-relational difficulties [45,46] and suffer bullying and teasing from peers [14,47,48]. 

Additionally, greater severity of NF1 is associated with more pronounced neuropsychological disorders, such as cognitive and learning difficulties [49], attention deficits [50], memory deficits [51] and intellectual disability [52]. These conditions can adversely affect school performance [25,53].

The relationship between QoL and NF1 severity was also confirmed by parents’ reports, but it was limited to the effect on school functioning. This result might suggest that the severity score measured by the modified Ablon scale [14], which accounts for a comprehensive list of neurodevelopmental issues, better delineates the neurological phenotype of patients. A worse severity score may be attributed to a more severe neurodevelopmental phenotype that might not be perceived by children. Moreover, school function reflects the adaptation profile of a child well, which is fundamental for parents. 

In conclusion, the results suggest that for patients, the disease primarily impacts physical and social QoL, whereas for parents, the main concern is school functioning.

However, there are some limitations to this study. Firstly, the limited number of participants suggests caution when generalizing the results. Despite this, few studies have investigated the relationship between QoL and the severity and visibility of NF1. 

Secondly, using a non-NF1-specific questionnaire for assessing QoL presented certain limitations. Specifically, the PedsQL scale employed in this study is not tailored to NF1. Consequently, we could not explore specific QoL dimensions pertinent to NF1, such as “Skin Sensations” and “Skin Itch Bother”, which are included in the PedsQL Neurofibromatosis Type 1 Module [24]. Regrettably, an adapted version of this module suitable for the Italian population is not yet available. 

The modified Ablon scale considered features such as dysmorphisms, short stature and scoliosis, which are relatively common in children with NF1 as related to visibility that was not included in the original scale. 

Using this version of the scale, we documented a significant correlation between visibility and severity, suggesting that visibility might be considered as an indirect index of disease severity. This novel finding must be further evaluated by future dedicated studies.

A further limitation is the lack of a subjective measure of the visibility and severity of the disease. Such measures could provide information about how the patients perceive and evaluate their condition. Ideally, using subjective tools alongside objective methods to measure the visibility and severity of the disease would be desirable to further explore the association between NF1 and QoL. Future studies should replicate these findings and attempt to address these limitations.

The findings of this study underscore the necessity for comprehensive QoL assessments that consider both visible and non-visible aspects of NF1. Clinicians should be encouraged to use multidimensional assessment tools that capture the full spectrum of the impact of NF1 on a child’s life. The early identification of QoL disorders could facilitate the implementation of proactive and personalised treatment plans, thereby improving patient outcomes. The association between NF1 visibility and social QoL suggests that psychosocial interventions aimed at reducing social stigma, strengthening self-esteem and improving relationships with peers could significantly benefit these patients. Support groups, counselling and social skills training could be valuable components of a comprehensive treatment plan.

## 5. Conclusions

The findings of this study highlight the intricate association between disease severity, visibility and specific domains of QoL in children and adolescents with NF1. 

This association suggests that tailored interventions are necessary to address the physical, social and academic challenges faced by patients with NF1. 

Future research should focus on conducting longitudinal studies to monitor changes in QoL over time in children and adolescents with NF1. This approach would provide valuable insights into the progression of the disease and its long-term impact on various QoL domains, thereby allowing for the development of timely and effective interventions.

In conclusion, our findings underscore the importance of conducting a comprehensive assessment of QoL in children with NF1, considering both patient and parent perspectives. By addressing the many challenges associated with NF1, future research and clinical practice can significantly improve the quality of life of these patients and ensure they receive the comprehensive support and care they need.

## Figures and Tables

**Table 1:** Comparison between the original Ablon scale [29] and the modified Ablon scale [14].

	Visibility				Severity		
	None	Mild	Moderate	Severe	Mild	Moderate	Severe
**Modified Ablon** **Scale**	No neurofibromas anywhere on the body; gait and posture unremarkable on casual inspection; no scoliosis or other abnormal skeletal features; no dysmorphisms.	No visible cutaneous and subcutaneous neurofibromas outside normal areas of clothing. However, it should be noted that many people who have no tumors in visible areas have numerous neurofibromas on the chest, abdomen, pelvis or thighs, which would be conspicuous in intimate situations and may severely affect sexual behavior; mild scoliosis or scoliotic posture or other skeletal features that are not perceived in impersonal interaction due to abnormal gait and posture.	Some neurofibromas on the neck, face, hand, forearm and leg or detectable in impersonal interaction because of modification of normal physical appearance; moderate scoliosis or other abnormal skeletal features visible in impersonal interaction and intimate situations; dysmorphisms are included in this class; short stature (<3°).	Numerous neurofibromas on the face; severe scoliosis or skeletal features with a noticeable limp; optic glioma that affects sight and eye socket.	Neurofibromas or mild learning and/or speech disorders that do not threaten physical or social life; mild scoliosis.	Numerous external and internal neurofibromas, moderate scoliosis, learning and/or speech disorders that compromise social interaction and lifestyle.	Neurofibromas that threaten function; serious internal neurofibromas; malignancies; optic glioma; severe scoliosis or other abnormal severe skeletal features.
**Ablon scale**	/	Essentially, no visible tumors outside of normal clothing areas; gait and posture appear unremarkable when casually observed (this allows for a heavy coating of neurofibromas on the body and some minor skeletal symptoms).	Some tumors on the neck, face. hands, mild scoliosis or other skeletal features without a noticeable limp.	Numerous tumors on the face, optic glioma that has affected sight and eye socket, severe scoliosis or skeletal features with noticeable limp.	Symptoms such as neurofibromas or mild learning disorders which do not threaten physical or social life.	Symptoms may compromise lifestyle but are not severely threatening. Numerous external or internal neurofibromas, mild scoliosis and controlled learning disorders.	Hundreds or thousands of visible neurofibromas which threaten functioning; blinding optic glioma; severe scoliosis or other skeletal features; serious internal neurofibromas; or malignancies.

**Table 2:** Comparisons between patients and parents results of QoL. Data are reported as median and (IQR).

	Patients	Parents	U Mann–Whitney	*p*-Value
Physical Functioning	5.00 (8)	4.00 (12)	685.000	0.699
Emotional Functioning	6.00 (7)	5.50 (7)	675.500	0.628
Social Functioning	1.00 (6)	4.00 (7)	547.000	0.065
School Functioning	6.00 (6)	6.00 (5)	612.500	0.335
QoL Total Score	77.00 (22)	73.50 (24)	696.000	0.787

## Data Availability

The authors declare that all data used in the conduct of the analyses are available within the article and tables and figures. To protect the privacy and confidentiality of patients in this study, clinical data are not publicly available in a repository or in the Supplementary Materials of the article, but they can be made available upon reasonable request to the corresponding author. Those requests will be reviewed by a study steering committee to verify whether the request is subject to any intellectual property or confidentiality obligations. All data shared will be de-identified.

## Supplementary Materials

The following supporting information can be downloaded at: https://www.mdpi.com/article/10.3390/children11081033/s1, Table S1: Comparison between the original Ablon scale (Ablon, 1996 [29]) and the modified Ablon scale version (Cavallo et al., 2023 [14]).
