# Supplementary material for: Quality of Life in Children with Neurofibromatosis Type 1: Agreement between Parents and Patients, and the Role of Disease Severity and Visibility
Source: Children (Basel). 2024 Aug 22;11(8):1033. doi: 10.3390/children11081033 (PMC11352328; doi:10.3390/children11081033)
Supplement: Supplementary file 1 [file children-11-01033-s001.zip › children-3145726-supplementary.pdf]

Supplementary Table S1. Comparison between the original Ablon scale (Ablon, 1996) and the modified Ablon scale version (Cavallo et al., 2023)

	Visibility				Severity		
	None	Mild	Moderate	Severe	Mild	Moderate	Severe
**Modified Ablon Scale**	No neurofibromas anywhere on the body; gait and posture unremarkable on casual inspection; no scoliosis or other abnormal skeletal features; no dysmorphisms.	No visible cutaneous and subcutaneous neurofibromas outside normal areas of clothing. However, it should be noted that many people who have no tumours in visible areas have numerous neurofibromas on the chest, abdomen, pelvis or thighs, which would be conspicuous in intimate situations and may severely affect sexual behaviour; mild scoliosis or scoliotic posture or other skeletal features that are not perceived in impersonal interaction due to abnormal gait and posture.	Some neurofibromas on the neck, face, hand, forearm, leg or detectable in impersonal interaction because of modification of normal physical appearance; moderate scoliosis or other abnormal skeletal features visible in impersonal interaction and intimate situations; dysmorphisms are included in this class; short stature (<3°).	Numerous neurofibromas on the face; severe scoliosis or skeletal features with a noticeable limp; optic glioma that affects sight and eye socket.	Neurofibromas or mild learning and/or speech disorders that do not threaten physical or social life; mild scoliosis.	Numerous external and internal neurofibromas, moderate scoliosis, learning and/or speech disorders that compromise social interaction and lifestyle.	Neurofibromas that threaten function; serious internal neurofibromas; malignancies; optic glioma; severe scoliosis or other abnormal severe skeletal features.
**Ablon scale**	/	Essentially no visible tumors outside of normal clothing areas; gait and posture appear unremarkable when casually observed (this allows for heavy coating of neurofibromas on the body and some minor skeletal symptoms).	Some tumors on neck, face. hands, mild scoliosis or other skeletal features without noticeable limp.	Numerous tumors on face, optic glioma (tumor) that has affected sight and eye socket, severe scoliosis or skeletal features with noticeable limp.	Symptoms such as neurofibromas or mild learning disorders which do not threaten physical or social life.	Symptoms may compromise lifestyle but are not severely threatening. Numerous external or internal neurofibromas, mild scoliosis, controlled learning disorders.	Hundreds or thousands of visible neurofibromas which threaten functioning; blinding optic glioma; severe scoliosis or other skeletal features; serious internal neurofibromas; or malignancies.
